# The health impact of an innovative summer camp for older adults: a pilot study using an interdisciplinary collaborative approach

**DOI:** 10.1186/s12912-021-00782-1

**Published:** 2022-01-04

**Authors:** Mei-Hua Yeh, Cheng-Hsien Huang, Yu-Chih Lin, Tung-Jung Huang, Mei-Yen Chen

**Affiliations:** 1 Department of Respiratory Therapy and Nursing, Chang Gung Memorial Hospital, 638, Yunlin, Taiwan; 2 Department of Family Medicine, Chang Gung Memorial Hospital, 638, Yunlin, Taiwan; 3 Department of Internal Medicine, Chang Gung Memorial Hospital, 638, Yunlin, Taiwan; 4 Department of Nursing, Chang Gung University of Science and Technology, 613, Chiayi, Taiwan; 5 School of Nursing, Chang Gung University, 333 Taoyuan, Taiwan; 6 Department of Cardiology, Chang Gung Memorial Hospital, 613, Chiayi, Taiwan; 7 No. 2, Chiapu Rd. West Sec, 613, Putz City, 61363 Chiayi County Taiwan, ROC

**Keywords:** Older adults, Innovative summer camp (ISC), Lung function test, Baduanjin, Health-related fitness

## Abstract

**Background:**

Healthy aging with dignity and aging in place are important for Taiwanese individuals. Although Taiwan did not experience COVID-19 outbreaks prior to May 2021, many older adults have been encouraged to stay at home since the beginning of the global pandemic in January 2020. Such shelter-in-place recommendations have resulted in significant lifestyle changes, limiting activities associated with aging with dignity such as exercise and community engagement. Few studies have explored how to promote or maintain holistic health practices and physical fitness in older adults residing in rural communities during the COVID-19 pandemic. This pilot study aimed to establish an interdisciplinary collaboration with community care workers (CCWs) and evaluate the impact of an innovative summer camp (ISC) program for older adults residing in rural areas.

**Methods:**

A quasi-experimental pre-post-test design with an interdisciplinary collaborative approach was implemented. The ISC program was based on a standardized protocol of modified Baduanjin exercise combined with three recreational breathing games. Participants were recruited from three community centers around the western coastal region of Yunlin County between June and August 2020. The ISC program was designed and executed by a nurse-led health promotion research team that collaborated with trained CCWs for 90 min per day, five days per week, for 12 weeks. Participants and CCWs wore facemasks during all activities. Paired *t*-test was used to measure changes in physical biomarkers, pulmonary lung function, and health-related fitness changes.

**Results:**

Sixty-eight participants completed the ISC program. The ISC program significantly improved the participants’ physiological biomarkers and health-related fitness, including reduced body weight, waistline, and systolic blood pressure, and increased forced vital capacity, biceps arm flexion, and ability to sit and stand from a chair, step with a knee up *in situ*, and stand on one foot with eyes open. Most participants reported that they felt happy, satisfied, and hoped that this program would be continued in their community center.

**Conclusions:**

This interdisciplinary, collaborative ISC program improved physical biomarkers and health-related fitness in older adults. Despite limitations, results strongly suggested that primary healthcare providers and CCWs can employ the described ISC program to promote wellness in older adults.

## Background

In Taiwan, the average life expectancy at birth is 81 years (male: 77.7; female: 84.2); however, the healthy life expectancy at birth is 71 years [1, 2]. This significant gap between life expectancy and healthy life expectancy means that nearly 10 years of life are spent disabled and with poor health before death. Healthy life expectancy is a useful indicator of a population’s overall health, reflecting the length of life as well as the quality of life. It also refers to an individual’s length of a life lived without limitations in conducting daily activities [3, 4]. Furthermore, in an aging society, as greater age puts increased pressure on social systems, extending a healthy life expectancy and shortening life expectancy with disability are becoming global priorities [5]. The ideal concepts of healthy aging with dignity and aging in their living place are gradually becoming a common ideology for most Taiwanese people and are included in all health policies [1, 2]. Since 2010, the government in response to the WHO’s initiative began the promotion of “age-friendly cities” suitable for older adults to live and work in peace [1]. Taiwanese government initiated a new version of a long-term care program, the mission called prevention disability and slowing aging. Under this program, any adult resident who receives a certificate for 90 h of training by nurses or another healthcare professionals can be certified as a community care worker (CCW), and a budget provided by the local government assists them in establishing community activity centers for older adults for social participation, physical activity, or free meals [2, 3]. To effectively establish these centers and related programs, many CCWs have requested instructional material concerning the design of interesting, simple, and beneficial health programs for older adults.

Previous studies have indicated that adopting regular exercise has health benefits including improved lung function and reduced incidence of most non-communicable diseases, especially in the aging population [6–9]. Aging is accompanied by changes in lung function due to factors such as loss of lung elasticity, weakened respiratory muscles, and decreased surface area for alveolar gas exchange. Accordingly, lung function parameters such as forced vital capacity (FVC) and forced expiratory volume in 1 s (FEV1) decline with age [9, 10]. Baduanjin (also called the eight-section brocade) is a popular exercise in Taiwan and is characterized by low-intensity aerobic exercise comprising of eight simple, safe, slow, coordinated, and sequential movements [11–13]. Baduanjin has been shown to significantly improve the physical and mental health of older adults by reducing lower back pain, symptoms of knee osteoarthritis, obesity, depression, blood pressure, waistline, frailty, and static and homeostasis, cardiopulmonary endurance [13–16]. Furthermore, the literature has indicated that certain social activities can positively affect health and well-being in older adults. For instance, frequent participation in karaoke, a group singing activity, has been shown to improve respiratory function in older adults and improve mood and reported sense of well-being [17–19].

Due to the global COVID-19 pandemic and subsequent shelter-in-place recommendations, many community-based activities have become limited, including group exercise and socialization. Related lifestyle changes have negatively impacted many individuals’ quality of life, including older adults. Although zero domestic COVID-19 cases were reported in Taiwan prior to May 2020 [20], older individuals were encouraged to stay home starting as early as January of 2020, preventing their participation in activities that benefit their health and well-being. As a result, many CCWs turned to our team, the health promotion nursing team for older adults at the Chang Gung University of Science and Technology College of Nursing, to instruct them how to initiate promotional health programs for the older adults in their communities during the COVID-19 pandemic. Nursing faculty were invited to speak at community activity centers to inform CCWs of best practices to avoid contagious disease transmission, enhancing lung function, and improving general health in aging individuals. However, few studies have explored how to promote or maintain holistic health practices and physical fitness in older adults residing in rural communities. Therefore, we sought to establish and evaluate a community-based, interdisciplinary approach to improve health and fitness in rural-dwelling older adults during the COVID-19 pandemic. To do so, we developed and executed an innovative, 12-week “summer camp” (ISC) program specifically tailored for older individuals in rural communities. The program was termed “summer camp” due to its resemblance to programs offering daily recreational games and activities over the summer period. We hypothesized that participation in this pilot ISC health promotion program would improve physical biomarkers, pulmonary lung function, and health-related fitness of older adults residing in rural communities.

## Methods

### Study design and participants

A single-group, quasi-experimental, pre-post-test study design was applied in three rural villages in western coastal Yunlin County. Originally, we sought to evaluate the efficacy of the ISC program by comparing ISC participants (treatment group) with a group of older adults who were not offered ISC participation (control group). After the conclusion of the three-month study period and data collection, we planned to offer the ISC program to the control villages. However, community leaders in the participating communities objected to control group participation and requested ISC program administration for their communities as well. Thus, a designated control group was not included in this study.

Participants were recruited from three rural community activity centers between June and August 2020. The nurse-led research team designed the ISC program and trained CCW collaborators prior to conducting the program. The ISC program comprised of a standardized protocol of modified Baduanjin exercise combined with three recreational breathing games. Typically, traditional Baduanjin consists of eight separate activities; however, due to the complexity three of these sections, we developed a modified Baduanjin exercise protocol that eliminated these sections and was shortened to five parts. In addition to participation in Baduanjin, older adults were provided with games, tools, and toys from their childhood and were offered recreational activities and lunch before returning home. Each week, the ISC program was designed and conducted by the research team. ISC programming lasted approximately 90 min, including 30 min of modified Baduanjin exercise, 30 min of table tennis, blowing games, and elephant-trunk paper flute (similar to a common pulmonary rehabilitation game), and 30 min of karaoke competition (a type of interactive entertainment developed in Japan in which participants sing along to recorded music using a microphone). Furthermore, we video recorded these sessions and shared ISC activities with other CCWs via YouTube in order to train them for the subsequent four days of activities each week. The inclusion criteria were as follows: (1) able to access the community center by walking or transportation (2) able to communicate in Mandarin or Taiwanese, and (3) agreed to participate in this study and signed the informed consent form.

### Measurements

1. *Demographic characteristics and health-related behaviors* included age, sex, education level, occupation, and living arrangement. Based on the previous studies [1–3], participants were asked questions regarding five health-related habits: (a) Cigarette smoking: “Do you smoke cigarettes?” Participants were classified as “non-smoker” if they reported having never smoked; “smoker” if they reported that they were current smokers or they smoked previously and had ceased smoking, (b) “Do you live with smokers?” Participants were categorized according to their response “yes” or “no.” For the following four items, responses were combined and categorized as never/seldom and usually/always: (c) Intake of vegetables: “How often do you consume three portions of vegetables (about 1.5 bowls) per day?“ (d) Intake of fruit: “How often do you consume two portions of fruit (about one bowl) per day?“ (e) Intake of water: “How often do you consume at least 1500 mL of water per day? (f) Regular exercise: “How often do you engage in exercise for at least 30 min, times per week?”
2. *Lung function test*s were performed by a certified respiratory therapist and experienced technician (MH, first author) using an automated flow-sensing spirometer (Pony Fx-EN13485, the new generation of desktop lung function tester developed by COSMED) based on the American Thoracic Society recommendations [9]. Three indices were frequently used by the clinicians to identify airway diseases: (1) forced expiratory vital capacity (FVC), which refers to the total amount of air that an individual can exhale in one breath, (2) predicted FVC value (%), and (3) forced expiratory volume in one second (FEV1)/FVC ratio (%) [10].
3. *Health-related fitness assessment* was based on measures for the implementation of National Fitness Testing by the Ministry of Sports Education for older adults. The assessment checklist primarily focused on evaluation of the participants’ cardiopulmonary function and physical fitness using the following metrics: (a) upper limb muscle strength and endurance (duration times of biceps arm flexion for 30 s); (b) leg muscle strength and endurance (duration times of sitting and standing for 30 s); (c) cardiorespiratory endurance (duration times of raising the knee and stepping on the spot for 2 min); (d) shoulder softness (back grasp test); (e) lower limb softness (sitting on the chair and bending forward); (f) static balance ability (standing on single foot with the eyes open); (g) physical agility and dynamic balance ability (standing from a chair, moving around objects, and returning to the chair). According to the standardized test by the Ministry of Sports Education [8], points were based on the participant’s sex and age, wherein values 1, 2, 3, 4, and 5 were considered very weak, weak, normal, good, very good, respectively. We assessed the health condition, including parameters such as blood pressure and pulse rate, prior to beginning the study program.

### Procedure and ethical consideration

 This study was performed in accordance with the Declaration of Helsinki and was approved by the institutional review board of the ethical committee of Chang Gung Memorial Hospital Foundation (IRB: 202000109B0). Participants who agreed to participate in the study provided informed consent in the form of a written consent form. Illiterate participants provided informed consent following oral presentation of the written consent form which was then signed by their guardian or companion following their permission to sign on their behalf.

 Before conducting the study, the research team (including one family physician, two nursing staff, one respiratory technician, one physical therapist, and five senior nursing students) conducted a general health assessment via a checklist (including measurement of the participants’ blood pressure and temperature, and asking questions such as: How are you feeling today? Did you have breakfast?) to determine whether he/she was eligible to participate in this program. The checklist was recorded and continued by a CCW for another four days per week. The study’s purpose and procedures were explained to all participants, three village leaders, and all CCWs. The village leader sent messages regarding the information and invited individuals to participate in this study. Upon agreeing, one-to-one measurement was conducted at each community activity center before and after this study.

The research team described the study procedures to all participants, including opening the windows, wearing surgical facemasks except when drinking water or participating in recreational breathing games, keeping social distance, the procedure of the lung function test, and health-related fitness [21, 22]. The ISC program was 90 min per day, five days a week for 12 weeks. On day 1, after the research team demonstrated the procedures, and each CCW followed the ISC protocol with the recorded video material for the other four days. Besides, the three CCWs were taught how to assist older adults in checking their blood pressure via the automated oscillometric monitor (Omega 1400; Orlando, Florida, USA) every morning, temperature, frequent hand hygiene, drinking water, and using the toilet during each session. During the post-test, we asked a general question to all participants “how do you feel/are you satisfied with this program?” The answer was descriptively recorded.

### Data analysis

Based on a two-tailed *t*-test (Cohen, 1992), the sample size was set at 55, which was calculated by the G^*^power 3.1.9.2 version, when the effect size = 0.4, α = 0.05, and power = 0.90. Due to the limited literature concerning similar interventions, we opted to use a medium effect size to calculate the sample size. Considering the 20% retraction rate, 82 participants were recruited for this study. The paired *t*-test was used to compare the mean difference in the physiological biomarkers (e.g., blood pressure, waistline, body mass index, three lung function parameters, and health-related fitness changes). Data analyses were conducted using SPSS 20 (IBM SPSS, Armonk, NY: IBM Corp).

## Results

### Demographic characteristics

The criteria were met by 85 older adults at the beginning of the study, of which 13 were excluded due to attending <70% of the program and four were excluded as they moved to a different county. Finally, 68 participants completed the study. The mean age of the participants was 73.9 years (standard deviation, 9.1). Most participants were women (66.2%), 47.1% were illiterate, and 36.8% were living alone (Table 1). We further compared the differences between participants who completed (51/68) and did not complete (17/68) the ISC program participants based on their demographic characteristics (age, sex, and educational level), pretest physiological biomarkers, lung function test, and health-related fitness. There was no significant difference between both groups, except the SKU. The mean steps were higher in the group that did not complete the ISC program than in those who completed the program (t=2.71, *p*<.01). Regarding health-related behaviors before conducting the program, 26.5%, 44.2%, 41.2%, and 55.8% reported that they infrequently consumed enough servings of vegetables, fruit, or water, or participated in exercise regularly, respectively. In addition, 17.7% of participants indicated that they lived with cigarette smokers.

**Table 1:** Demographic characteristics and health-related behaviors (*N*=68)

Variables	N (%)			
Gender				
Female	45 (66.2)			
Male	23 (33.8)			
Age (years)	Mean=73.9; SD=9.1; Range 65~90			
Education level (years)				
Illiterate	32 (47.1)			
≦ Middle school	27 (39.8)			
≧ High school	9 (13.3)			
Marital status		Intake vegetable ≥ 3 portions per day		
Married/ divorce	45 (64.7)	Never/seldom	18 (26.5)	
Widowed/single	23 (33.8)	Usually/always	50 (73.5)	
Living arrangement		Intake fruit 2 portions per day		
Alone	25 (36.8)	Never/seldom	30 (44.2)	
Lived with others	43 (63.2)	Usually/always	38 (55.8)	
Occupation		Intake of water >1500 mL per day		
No	45 (66.2)	Never/seldom	28 (41.2)	
Yes	23 (35.8)	Usually/always	40 (58.8)	
Cigarette smoking		Adopting regular exercise>30 min ≧3 times		
Never	59 (86.8)	Never/seldom	38 (55.8)	
Current /former users	9 (13.2)	Usually/always	43 (44.1)	
Living with smoker				
No	56 (82.4)			
Yes	12 (17.6)			

### Changes in physiological biomarkers and health-related fitness before and after the ISC program

Table 2 presents the participants’ physiological biomarkers after the three-month intervention. The body weight (t=2.19, *p*<.05), waist circumference (t=3.97, *p*<.001), systolic blood pressure (t=4.01, *p*<.001), forced vital capacity (t= -4.44, *p*<.001); forced vital capacity predicted (t=-4.09, *p*<.001), and forced expiratory volume in 1 s predicted (t=-4.51, *p*<.001) significantly improved. As shown in Table 3, participant’s biceps arm flexion (t= -3.01, *p*<.01), sit and standing from a chair (t=-4.99, *p*<.001), stepping with a knee up in situ (t=-4.31, *p*<.001), and standing on one foot with the eyes open (t=-2.57, *p*<.05) were significantly increased after the three-month intervention.

**Table 2:** Physiological biomarkers changed before and after the summer camp program (*N*=68)

Variables	Before After Mean (SD)		*t*	*P*	95% CI*
Body weight	63.91 (11.38)	63.18 (10.94)	2.19	<0.05	0.06~1.39
Body mass index	25.97 (3.86)	25.98 (3.87)	-0.02	0.983	-0.44~0.43
WC^1^	90.51 (9.34)	88.68 (8.42)	3.97	<0.001	0.91~2.76
SBP^2^	138.82 (16.97)	128.65 (22.25)	4.01	<0.001	5.11~15.24
DBP^3^	72.85 (12.86)	73.69 (12.31)	-0.57	0.572	- 3.78~2.11
Pulse rate	78.34 (11.80)	77.99 (12.11)	0.36	0.720	-1.61~2.31
FVC (L)^4^	1.98 (0.70)	2.19 (0.76)	-4.44	<0.001	- 0.30~- 0.11
FVC % predicted (L)^5^	76.44 (18.05)	83.38 (15.97)	-4.09	<0.001	-10.33~- 3.55
FEV1 predicted (%)^6^	75.91 (18.15)	84.19 (15.05)	-4.51	<0.001	-11.94~- 4.62

*Confidence interval; ^1^WC, waist circumference; ^2^SBP, systolic blood pressure; ^3^DBP, diastolic blood pressure; ^4^FVC, forced vital capacity; ^5^FVC%, forced vital capacity predicted; ^6^FEV1, forced expiratory volume in 1 s predicted

 When we asked each participant the general question, “how do you feel/ are you satisfied with this program?,” 97.1% (*n*=66) of participants responded positively that they were happy and satisfied with the program (e.g., “I expect to come here again to see all of my friends… It is fun to play with friends… I love the interesting classes so much… I’m so happy when I sweat during exercise… I felt happy that I could sleep well every day after classes… I can learn much useful health education from all of you… I hope this class can be continued forever.”). Only two participants complained, stating “I felt uncomfortable when wearing a facemask during exercise…” Furthermore, three CCWs also gave positive feedback, including “Thank you so much for teaching us how to handle the program… the recreational games are so funny and are not boring to the older individuals… so… they love coming here… I felt they were happier than before… hope your team continues to support our village…”.

**Table 3:** Health-related fitness changed before and after the summer camp program (*N*=68)

Variables	Before After Mean (SD)		*t*	*P*	95% CI*
BAF (time/30 seconds)^1^	15.32 (5.79)	16.54 (6.11)	-3.01	0.004	- 2.03~ - 0.41
SSC (time/30 seconds)^2^	12.88 (5.47)	14.38 (5.79)	- 4.99	<0.001	-2.10~ -0.90
SKU (time/120 seconds)^3^	106.43 (48.25)	122.40 (52.59)	-4.31	<0.001	- 23.36~ -8.58
BST (cm)^4^	14.32 (15.27)	12.12 (16.56)	1.57	0.121	-4.99~0.60
SFB (cm)^5^	0.92 (11.41)	0.79 (13.17)	0.11	0.912	-2.20~ 2.46
SOF (seconds)^6^	10.58 (10.94)	11.86 (12.19)	-2.57	0.012	- 3.14~ -0.39
GCB (seconds)^7^	11.37 (6.20)	12.46 (14.27)	- 0.66	0.512	- 4.38~ 2.20

*Confidence interval; 1BAF, biceps arm flexion (assess upper limbs muscle endurance); 2SSC, sit and stand from chair (assess leg muscle endurance); 3SKU, step with knee up in situ (assess cardiopulmonary function and aerobic fitness); 4BST, back scratch test (assess shoulder softness); 5SFB, sitting forward bending (assess lower limbs softness); 6SOF, stand on one foot with eyes open (access static balance); 7GCB, get up from the chair, and bypass items, then return back to seat (access physical agility and dynamic balance).

## Discussion

This pilot study explored the effectiveness of an innovative, interdisciplinary ISC program that combined modified Baduanjin exercise and three recreational breathing games for older adults in rural communities. Although this study has some limitations with respect to the study design, the present findings indicate that after twelve weeks of participation in the ISC program, lung function parameters and health-related fitness were significantly improved. Thus, this pilot study suggests that similar programs may be effective in improving physical health and wellbeing in older adults residing in rural communities. However, due to the lack of a control group and the relatively small sample size, further studies are necessary confirm these results.

The present findings are similar to results of previous studies that demonstrate that sequential movements of modified Baduanjin exercise, blowing games, and karaoke competition have beneficial health effects including reducing obesity, waist circumference, and systolic blood pressure, and improving lung function and feelings of happiness [15–19]. Previous studies have shown that decreased pulmonary function was found to be associated with low muscle mass in the Korean community-dwelling older adults. Moreover, forced expiratory volume in 1 s and forced vital capacity were positively correlated with appendicular skeletal muscle [6]. Although we did not measure sarcopenia or muscle mass change in this program, the short three-month intervention had significantly improved lung function parameters. The possible reasons might be due to the ISC program which enhanced both upper and lower limb muscle endurance and maintained the cardiopulmonary function via the sequential movements of modified Baduanjin and the recreational breathing games. Maintaining adequate muscle mass is very important for older adults to prevent decline in lung function and physical wellbeing. Further randomized control studies are needed to evaluate the change in muscle mass after participating in the ISC program. After participation in the 12-week ISC program, we found that three physiological biomarkers (body mass index, diastolic blood pressure, and pulse rate) remained unchanged. It is possible that these biomarkers remained unchanged due to the relatively short period of intervention, or that these three biomarkers are less suitable as outcome evaluators for the aging population.

The present findings were similar to those of a study conducted in Japan by Miyazaki and Mori [17] that found that frequent karaoke training for older adults significantly improved their bedside Frontal Assessment Battery scores compared to that of an active control group receiving scratch art training. The karaoke intervention group also demonstrated improved tongue pressure and pulmonary function, with a greater increase in FEV1. Miyazaki and Mori also noted that frequent karaoke appears beneficial for slowing cognitive decline and preventing dysphagia by sarcopenia. Further, studies conducted in Norway by Batt-Rawden et al. and Stedje et al. [18, 19] reported that singing serves as a health-promoting activity in care settings for aging individuals, and that singing could potentially reduce the need for medications and occurrence of accidents among nursing home residents. Although we did not assess cognitive function in this study, almost all participants responded positively to this program, reporting that they felt happy and enjoyed participating in the program. Further studies could explore some unhealthy behaviors which were assessed before conducting the program, including infrequently adopting adequate servings of vegetables, fruit, and water.

Although some studies had challenged the safety and efficacy of wearing facemasks as a preventive measure for COVID-19 [23–25], all participants in the present study followed the recommended guidelines by wearing surgical masks and maintaining social distance during participation in the ISC program. No study participants became infected with COVID-19 over the duration of the study. Although there have been zero domestic cases of COVID-19 in Taiwan since October 2021, and 77.9% (nearly 23 million) and 47.7% of the Taiwanese population (nearly 23 million) have received the first and second doses of the COVID-19 vaccination, respectively, our research team strongly suggested that the ISC program should follow COVID-19 prevention measures. Thus, the ISC program was designed to be executed in community centers while following the COVID-19 prevention measures (including 2 doses of vaccination, open windows with good ventilation, maintaining a social distance of 2 m to avoid airborne transmission, wearing face masks, and maintaining appropriate personal hygiene).

## Limitations

The present study had some limitations. First, the absence of a control group and the potential threats to the internal validity of the instrumentation must be considered, as these might limit the generalization of the findings. Thus, future studies must include a control group. Second, the sample size was small, and the population selection was not entirely random because all participants belonged to three communities from a single county. Third, the potential for a social desirability response bias also needs to be taken into consideration.

## Conclusions

This pilot study with an interdisciplinary collaborative approach supports the benefits of this simple, low-cost ISC program in improving physiological biomarkers, health-related fitness, subjective feeling of happiness, and satisfaction in older adults residing in rural communities. During the execution of the ISC program, no one was infected by COVID-19, suggesting these activities are appropriate despite modified safety precautions due to the COVID-19 pandemic. However, it is necessary to conduct further studies with both experimental and control groups to further evaluate these findings.

## Data Availability

The datasets used and/or analyzed during the current study are available from the corresponding author upon reasonable request.

## Abbreviations

ISC: innovative summer camp
WC: waist circumference
SBP: systolic blood pressure
DBP: diastolic blood pressure
FVC: forced vital capacity
FVC%: forced vital capacity predicted
FEV1: forced expiratory volume in 1 s predicted
BAF: biceps arm flexion
SSC: sit and stand from a chair
SKU: step with a knee up in situ
BST: back scratch test
SFB: sitting forward bending
SOF: stand on one foot with eyes open
GCB: get up from the chair, and bypass items

## References

[1] Health Promotion Administration, HPA. Healthy aging. Available online: https://www.hpa.gov.tw/EngPages/List.aspx?nodeid=4105. (Accessed 10 Feb 2021.

[2] Health Promotion Administration, HPA. Statistics of health promotion -2018, annual report of health promotion. Available online: https://www.hpa.gov.tw/Home/Index.aspx. Accessed 10 Feb 2021.

[3] Ministry of Health and Welfare, Taiwan. Long term care 2.0. Available from: https://1966.gov.tw/LTC/cp-3636-42415-201.html. Accessed 1 Feb 2021.

[4] Vos T, Allen C, Arora M (2015). Global, regional, and national incidence, prevalence, and years lived with disability for 310 diseases and injuries, 1990–2015: A systematic analysis for the global burden of disease study. Lancet.

[5] Hosokawa R, Ojima T, Myojin T (2020). Associations between healthcare resources and healthy life expectancy: a descriptive study across secondary medical areas in Japan. Int. J. Environ. Res. Public Health.

[6] Jeon YK, Shin MJ, Kim MH (2015). Low pulmonary function is related with a high risk of sarcopenia in community-dwelling older adults: the Korea national health and nutrition examination survey 2008-2011. Osteoporos. Int.

[7] Jacob ME, Yee LM, Diehr PH (2016). Can a healthy lifestyle compress the disabled period in older adults?. J. Am. Geriatr. Soc..

[8] Wu MC, Jane CF. Evaluation on the functional fitness of the elderly in Taiwan. Sports Administration, Ministry of Education. Available online: https://www.sa.gov.tw/. Accessed 12 Mar 2020.

[9] Gao C, Zhang X, Wang D (2018). Reference values for lung function screening in 10- to 81-year-old, healthy, never-smoking residents of Southeast China. Medicine..

[10] Thomas ET, Guppy M, Straus SE (2019). Rate of normal lung function decline in ageing adults: a systematic review of prospective cohort studies. BMJ Open..

[11] Li H, Ge D, Liu S (2019). Baduanjin exercise for low back pain: A systematic review and meta-analysis. Complement Ther Med.

[12] Li R, Chen H, Feng J, Xiao Y, Zhang H, Lam CW, Xiao H (2020). Effectiveness of traditional Chinese exercise for symptoms of knee osteoarthritis: a systematic review and meta-analysis of randomized controlled trials. Int J Environ Res. Public Health.

[13] Lin PC, Chen KT, Cheng YA, Tang RY, Tsai CC (2020). A study on the impact of Ba Duan Jin exercise on the physical and mental health of middle-aged and elderly people in the community. Journal of Chinese Medicine.

[14] Yang Q, Yu S, Wang J, Zheng C, Liang X, Yu D, Chen X (2021). Effects of Baduanjin on patients with chronic nonspecific low back pain: A randomized controlled trial. Medicine (Baltimore).

[15] Liu XY, Gao J, Yin BX, Yang XY, Bai DX (2016). Efficacy of Ba Duan Jin in improving balance: A study in Chinese community-dwelling older adults. J Gerontol Nurs.

[16] Zou L, Yeung A, Quan X (2018). Mindfulness-based Baduanjin exercise for depression and anxiety in people with physical or mental illnesses: A systematic review and meta-analysis. Int J Environ Res Public Health.

[17] Miyazaki A, Mori H (2020). Frequent karaoke training improves frontal executive cognitive skills, tongue pressure, and respiratory function in elderly people: pilot study from a randomized controlled trial. Int J Environ Res Public Health.

[18] Batt-Rawden KB, Stedje K (2020). Singing as a health-promoting activity in elderly care: a qualitative, longitudinal study in Norway. J Res Nurs.

[19] Batt-Rawden KB, Andersen S (2019). Singing has empowered, enchanted and enthralled me choirs for wellbeing?. Health Promot Int.

[20] Taiwan Center for Disease Control, CECC. Attention COVID-19. Available online: https://www.cdc.gov.tw/En

[21] Su YF, Yen YF, Yang KY, Su WJ, Chou KT, Chen YM, Perng DW (2020). Masks and medical care: Two keys to Taiwan’s success in preventing COVID-19 spread. Travel Med Infect Dis..

[22] Taiwan Centers for Disease Control, CDC. CECC reports no new confirmed cases; 420 patients released from isolation. Taiwan CDC. Available online: https://www.cdc.gov.tw/En/Bulletin/Detail/6NK4EDQ1Er0L74qZzNptkQ?typeid=158. Accessed 28 May 2020.

[23] Lo A, Huang JJ, Chen CC, Chou FH, Shieh VJ (2020). From biological safety to social safety: How Taiwan’s community centered prevention program controlled the COVID-19 outbreak. Glob Health.

[24] Brainard J, Jones NR, Lake IR, Hooper L, Hunter PR (2020). Community use of face masks and similar barriers to prevent respiratory illness such as COVID-19: A rapid scoping review. Euro Surveill..

[25] Chaabna K, Doraiswamy S, Mamtani R, Cheema S (2020). Facemask use in community settings to prevent respiratory infection transmission: A rapid review and meta-analysis. Int J Infect Dis.

